# Impact of SARS-CoV-2 Preventive Measures against Healthcare-Associated Infections from Antibiotic-Resistant ESKAPEE Pathogens: A Two-Center, Natural Quasi-Experimental Study in Greece

**DOI:** 10.3390/antibiotics12071088

**Published:** 2023-06-22

**Authors:** Emmanouil Bolikas, Eirini Astrinaki, Evangelia Panagiotaki, Efsevia Vitsaxaki, Stamatina Saplamidou, Ioannis Drositis, Dimitra Stafylaki, Georgios Chamilos, Achilleas Gikas, Diamantis P. Kofteridis, Evangelos I. Kritsotakis

**Affiliations:** 1 Laboratory of Biostatistics, School of Medicine, University of Crete, 71003 Heraklion, Greece; 2 Infection Control Committee, Venizeleio-Pananeio General Hospital, 71409 Heraklion, Greece; 3 Infection Control Committee, University Hospital of Heraklion, 71110 Heraklion, Greece; 4 Department of Clinical Microbiology, Venizeleio-Pananeio General Hospital, 71409 Heraklion, Greece; 5 Department of Medical Oncology, Venizeleio-Pananeio General Hospital, 71409 Heraklion, Greece; 6 Department of Clinical Microbiology and Microbial Pathogenesis, University Hospital of Heraklion, 71110 Heraklion, Greece; 7 Department of Internal Medicine, School of Medicine, University of Crete, 71003 Heraklion, Greece

**Keywords:** antibiotic resistance, healthcare-associated infection, SARS-CoV-2, infection control, multidrug resistance, epidemiology

## Abstract

The COVID-19 pandemic led to unprecedented stress on healthcare systems worldwide, forming settings of concern for increasing antimicrobial resistance. We investigated the impact of SARS-CoV-2 preventive measures against healthcare-associated infections (HAIs) from antibiotic-resistant bacteria in two tertiary-care hospitals. We compared infection rates between March 2019 and February 2020 (pre-intervention period) and March 2020 and February 2021 (COVID-19 intervention period) from drug-resistant ESKAPEE bacteria (methicillin-resistant *Staphylococcus aureus*; vancomycin-resistant *Enterococci*; carbapenem-resistant *Klebsiella pneumoniae*, *Acinetobacter baumannii*, *Pseudomonas aeruginosa*, *Enteroba*cter species and *Escherichia coli*). Over 24 months, 586 drug-resistant ESKAPEE HAIs occurred in 439 patients (0.3% of 179,629 inpatients) with a mean age of 63 years, with 43% being treated in intensive care units (ICUs), and having a 45% inpatient mortality rate. Interrupted time series analysis revealed increasing infection rates before the intervention that were sharply interrupted by abrupt drops for most pathogens and henceforth remained stable in the ICUs but progressively increased in ordinary wards. In the ICUs, the pooled infection rate was 44% lower over the intervention period compared to the pre-intervention period (incidence rate ratio (IRR) 0.56, 95%CI 0.41–0.75, *p* < 0.001). Pooled infection rates in the wards were slightly higher over the COVID-19 period (IRR 1.12, 95%CI 0.87–1.45, *p* = 0.368). The findings confirmed the ancillary beneficial impact of the enhanced bundle of transmission-based precautions adopted against SARS-CoV-2 in rapidly constraining antimicrobial-resistant HAIs in two Greek hospitals.

## 1. Introduction

Bacterial antimicrobial resistance (AMR) poses a serious threat to global public health and was linked to an estimated 1.27 million deaths globally in 2019 [1]. Particularly concerning in healthcare settings are the ESKAPE bacteria (*Enterococcus faecium*, *Staphylococcus aureus*, *Klebsiella pneumoniae*, *Acinetobacter baumannii*, *Pseudomonas aeruginosa* and *Enterobacter* species) that frequently carry resistance mechanisms, allowing them to “escape” the biocidal effects of last-line antibiotics [2,3]. ESKAPE pathogens together with *Escherichia coli* (hereafter referred to as ESKAPEE) were responsible for more than 80% of global deaths attributable to AMR in 2019 [1]. Notably, carbapenem-resistant (CR) *A. baumannii*, *P. aeruginosa* and *Enterobacterales*; vancomycin-resistant Enterococci (VRE); and methicillin-resistant *S. aureus* (MRSA) were highlighted as multidrug-resistant pathogens of critical priority by the World Health Organization, which called for urgent research and antibiotic development for these pathogens [4].

AMR and the Coronavirus Disease 2019 (COVID-19) pandemic comprise two intersecting global public health crises. The latter formed a setting of major concern for increasing AMR by disrupting healthcare systems and practices all over the world [5,6]. Indeed, the US Centers for Disease Control and Prevention reported an increase in healthcare-associated infections (HAIs) caused by antibiotic-resistant ESKAPEE organisms during the first year of the pandemic [7]. In addition, a cohort study in 148 HCA healthcare-affiliated hospitals observed that COVID-19 surges adversely correlated with rates of MRSA bacteremia as well as catheter-related bloodstream infections and urinary tract infections [8]. A systematic review of 30 studies (up to July 2022) concluded increases in the rates of infection or colonization by antibiotic-resistant bacteria occurred during the COVID-19 pandemic, but it also noted paucity of data from several regions around the globe [9]. In contrast, another meta-analysis of 23 studies conducted around the same time (up to June 2022) found that the COVID-19 pandemic situation was not associated with changes in Gram-positive AMR rates but may have increased Gram-negative AMR, particularly in settings where infection prevention and control (IPC) and/or antimicrobial stewardship initiatives were not enhanced [10].

The relation between the contexts of AMR and COVID-19 is complex, as opposing effects from several factors may come into play. On one hand, pressured hospital systems, the exhaustion of healthcare professionals and shortages of personal protective equipment (PPE) during the pandemic may have facilitated transmission routes and the spread of antibiotic-resistant pathogens [5,6,7,9,11,12]. On the other hand, a natural experiment took place during the COVID-19 pandemic when transmission-based precautions and protective measures usually implemented in the ICU were further heightened and expanded in ordinary wards, which could have thwarted the effect of nosocomial AMR infections [10,13]. Empirical evidence to disentangle these effects is imperative for the healthcare system in Greece, which suffers from the high prevalence of AMR in acute care hospitals, with an estimated >25,000 patients annually in the country affected by difficult-to-treat HAIs caused by multidrug-resistant pathogens [14].

Against this background, we investigated whether enhanced infection prevention and transmission-based precautions adopted during the COVID-19 period for preventing the spread of SARS-CoV-2 might have also modified the incidence of antibiotic-resistant ESKAPEE-associated HAIs in two tertiary care hospitals in Greece.

## 2. Results

### 2.1. Affected Patients

Over the 24-month study period, we identified 439 patients (335 in Hospital A and 104 in Hospital B) with at least one episode of antibiotic-resistant ESKAPEE-associated HAI during their hospital stay. The affected patients had a mean age of 63 ± 21 years, 67% were male, 33% had a Charlson comorbidity index (CCI) ≥ 1, and 43% were treated in the ICU at the time of index infection onset. The mean length of hospital stay (LOS) before the onset of the index infection was 20 days. After infection onset, the 14-day and overall inpatient fatality proportions were 21% and 45%, respectively. The clinical characteristics and outcomes of affected patients did not differ significantly between the two study hospitals (Supplementary Material Table S2). There were 20 patients (5%) admitted with severe COVID-19 requiring critical care, who were elderly (mean age 73 ± 11 years) with a high inpatient mortality rate (17/20; 85%). 

When the characteristics of affected patients were compared between the pre COVID-19 and COVID-19 periods (Table 1), similar epidemiological features of antibiotic-resistant ESKAPEE HAIs were found. In particular, the distributions of patient sex, admission diagnosis, CCI, department of hospitalization, pre-index infection LOS, occurrence of multiple infections and overall LOS were similar between the two periods. The exception was patient age, which was greater during the COVID-19 period compared to the pre-pandemic period (mean 60 vs. 66 years, *p* = 0.002). Inpatient mortality also appeared to increase during the COVID-19 period (49% vs. 43%), but this difference was not statistically significant (*p* = 0.21) and reflected the high mortality of patients admitted with severe COVID-19. When the COVID-19 patients were excluded from analysis, the overall inpatient mortality (45% vs. 43%, *p* = 0.68) was much more similar between the two study periods. These results were consistent in subgroup analysis by source hospital (Supplementary Table S3). 

### 2.2. Before–After Pooled Infection Rates

As shown in Table 2, the distributions of infection sites and pathogens were largely similar between the study periods. Of note, proportionally fewer infections from CR *P. aeruginosa* and CR *K. pneumoniae* occurred during the pandemic period, but a higher percentage of hospital-acquired pneumonia cases was noted. However, the pre–post differences in the relative proportions of infection sites and pathogens varied by source hospital (Supplementary Material Table S4).

Small and statistically non-significant increases were observed in the COVID-19 period compared to the pre-COVID-19 period for both the pooled average incidence of infected patients (0.82 vs. 0.80 cases per 1000 patient-days; IRR = 1.03, *p* = 0.78) and the incidence of infections (1.11 vs. 1.06 infections per 1000 patient-days; IRR = 1.05, *p* = 0.58). However, there was variation by source hospital when the pre- and post-intervention rates were examined in subgroups the by site of infection and pathogen (Supplementary Material Table S5).

### 2.3. Temporal Trends and Level Changes in Infection Rates

**Figure 1:**
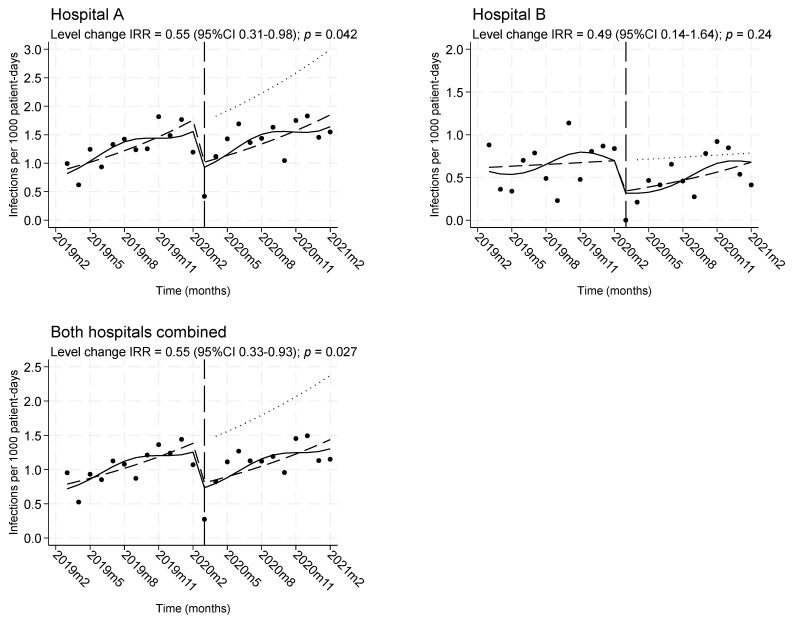
Monthly rates of healthcare-associated infections from multidrug-resistant ESKAPEE pathogens, before and during the intervention. Dots: observed rates. Solid line: predicted rates from a Poisson segmented regression model adjusted for seasonality. Dashed line: deseasonalized trend. Dotted line: counterfactual scenario assuming the intervention was not implemented. Vertical long dashed line: time of the beginning of the intervention.

The investigation of longitudinal trends via interrupted time series (ITS) analysis revealed an increasing trend of the overall infection rate during the pre-COVID-19 period, which was abruptly interrupted immediately after the intervention by a level decrease of −45% (IRR 0.55, 95%CI 0.33–0.93, *p* = 0.027). However, this was followed again by a progressive increase in infection rates during the COVID-19 period. As seen in Figure 1, similar trends were observed for both hospitals.

Level decreases occurred for all major pathogens and sites of infection, except urinary tract infections and VRE infections in Hospital A, but the subgroup numbers were too small and IRR estimates too uncertain for specific pathogen species (Supplementary Material Table S6). A notable level drop occurred for the rate of CR *K. pneumoniae* (IRR 0.14, 95%CI 0.03–0.69) immediately after the intervention.

### 2.4. ICUs vs. Wards

**Figure 2:**
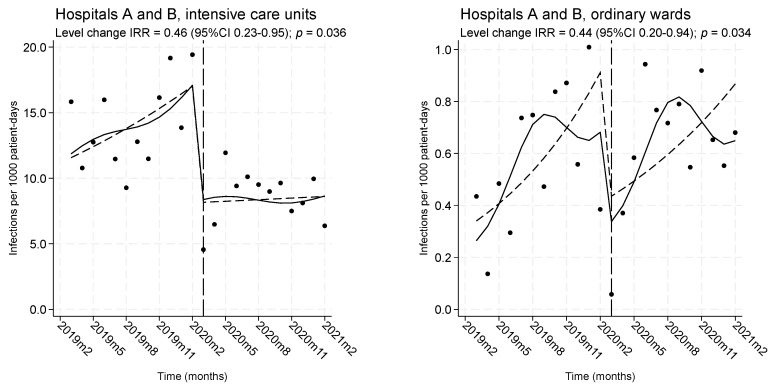
Monthly rates of healthcare-associated infections from multidrug-resistant ESKAPEE pathogens, before and during the intervention, presented separately for intensive care units and ordinary wards. Dots: observed rates. Solid line: predicted rates from a Poisson segmented regression model adjusted for seasonality. Dashed line: deseasonalized trend. Vertical long dashed line: time of the beginning of the intervention.

In the ICUs, the pooled average infection rate over the entire intervention period was reduced by 44% compared to the pre-intervention period (5.7 vs. 10.1 infections per 1000 ICU-days; IRR = 0.56, 95%CI 0.41–0.75, *p* < 0.001). In contrast, in the wards, the average infection rates were similar over the two periods (0.51 vs. 0.46 patient-days; IRR = 1.12, 95%CI 0.87–1.45, *p* = 0.368). As seen in Figure 2, significant level drops in overall infection rates occurred in both the ICUs and the ordinary wards immediately after the intervention. In both settings, increasing trends of infections during the pre-intervention period were sharply interrupted by the intervention and abruptly dropped. However, the level drops were followed by different trends during the COVID-19 period; a stable trend was noted in the ICUs, whereas an increasing trend was noted in the wards. 

## 3. Discussion

The key finding of this study is that the bundle of enhanced IPC measures adopted during the COVID-19 period for preventing the spread of SARS-CoV-2 had an ancillary beneficial impact by rapidly reducing the incidence of antibiotic-resistant ESKAPEE HAIs in two tertiary-care hospitals in Greece with varied practices, operational capacities and resources. In both hospitals, an underlying trend of increasing infection incidence during the 12 months before the intervention was abruptly interrupted after the beginning of the intervention via a relative reduction in the infection rate by about 45%, which was then followed by a stable trend in the ICUs but an increasing trend in ordinary wards. This led to a significant overall decrease in the pooled rate of infections by 44% in the ICUs and a statistically non-significant increase by about 12% in the wards over the 12-month COVID-19 intervention period relative to the 12-month pre-intervention period. 

To the best of our knowledge, this is the first comprehensive evaluation of the impact of SARS-CoV-2 preventive measures against AMR within the Greek public healthcare system. We are only aware of a single related study conducted in nine Greek hospitals that examined trends of the antimicrobial non-susceptibilities of major nosocomial pathogens before and after the changes due to COVID-19 and found varying trends depending on the bug–drug combinations [15]. However, that study relied on examining the proportions of clinical isolates that were antibiotic resistant (non-susceptible) and did not account for between-hospital heterogeneity [15]. Although non-susceptibility proportions are helpful for guiding empirical antibiotic therapy, proportion-based analyses can be misleading regarding the effectiveness of infection control interventions [16]. Several studies have illustrated that it is not reasonable to expect that changes in non-susceptibility proportions of pathogenic organisms reflect changes in the same magnitude and direction in the absolute frequency of infections from these organisms [17]. This is because factors influencing the susceptible bacterial population may affect the proportion of resistant isolates without necessarily changing their absolute number, and thereby without changing the burden of AMR [16]. We therefore utilized a more appropriate measure of AMR burden in this study, namely the incidence density rate of infections from antimicrobial-resistant ESKAPEE organisms, that is, the number of resistant isolates in the hospital population over time (expressed per patient-day). We also considered between-hospital variability in all analyses performed in this study, confirmed that there was no change in the case-mix of affected patients and relied on an ITS design to account for underlying temporal trends before comparing between the pre- and post-intervention periods [18,19,20,21]. 

A previous cross-sectional investigation conducted in April 2022 in all eight public acute-care hospitals in Crete (including the two hospitals in the present study) provided indirect evidence that the prevalence of HAIs, albeit relatively high, remained constrained during the enormous pressure on the hospitals from COVID-19 pandemic [22]. The present study provides high-level evidence that there was a concomitant benefit of the COVID-19 prevention measures on the incidence density of antibiotic-resistant HAIs in two of those hospitals. Although systematic literature reviews have been largely inconclusive on this matter [9,10], positive impacts on the rates of HAIs from applying strategies originally designed to contain SARS-CoV-2 have been observed in diverse hospital settings. In four community hospitals in Los Angeles, USA, the increased usage of alcohol sanitizer and hand soap among healthcare workers in the second quarter of 2020 (when COVID patients began arriving) coincided with decreases of 20% for extended-spectrum β-lactamase-producing organisms, to 41% for MRSA and 80% for VRE, relative to the first quarter of 2020 (pre-COVID-19) [23]. Similarly, a collateral benefit of the COVID-19 prevention measures, including measurable increases in alcohol for hand hygiene and surgical masks, was associated with a significant decrease in the incidence density of CR *A. baumannii* and VRE in a 1700-bed medical center in Taiwan [24]. Likewise, the containment measures implemented during the COVID-19 emergency, such as the mandatory use of surgical masks and restrictions on visitors, were associated with a reduction in surgical site infections in a surgical ward in Trieste, Italy [25]. Similarly, another longitudinal investigation in a surgical ICU in Rome, Italy, concluded that robust adherence to SARS-CoV-2 preventive measures was associated with a reduction in the frequency of multidrug-resistant ESKAPEE isolates in that unit [13].

It should be noted that not all circumstances allow for the possibility of increasing the number and stringency of IPC measures, especially when inpatient capacity and high workloads have been reached [26]. A rapid decline in the overall infection rate by 56% immediately after the intervention was observed in the hospital wards in this study, but this was not sustained and was followed by a progressive increase during the COVID-19 period. This is a reminder that balancing between adherence with strict IPC measures and other immediate clinical demands can be challenging. Substantial investments to redesign and additional institutional capacity may be necessary if IPC gains are to be sustained in the post-pandemic era [27]. 

With this study, we exploited the unique opportunity from a natural experiment that allowed us to assess the impact of implementing an enhanced bundle of hospital IPC measures during the COVID-19 period. However, an important limitation is that we could not disentangle the contributions of individual components of the intervention. It is possible that some intervention components might have made limited contributions to reducing the incidence of HAIs or even may have had negative impacts on other aspects of healthcare. For example, there are concerns of an overall negative impact from delaying elective surgeries [28] and from applying strict visitor restrictions on healthcare workers, patients and their families [29]. 

Likewise, it is difficult to disentangle the effect of the carbapenem-focused stewardship program that was implemented in Hospital A. This component of the intervention bundle was previously shown to have effectively reduced the use of carbapenems without increasing the consumption of newer antibiotics [30] and led to an improved quality of prescribing and improved patient outcomes. We can only speculate that the stewardship component contributed to the decreases in HAI incidence from CR Gram-negative pathogens observed in Hospital A over the COVID-19 period, especially for CR *K. pneumoniae* and CR *P. aeruginosa*. However, substantial decreases in the incidence of Gram-negative infections were also noted in Hospital B (especially regarding CR *A. baumanni*i), where the antibiotic policy remained unaltered over the entire study period. Nevertheless, we should consider that recent research on the ecological long-term effect of antibiotic use on AMR has demonstrated that a decrease in usage only slowly decreases AMR and the reduction is insubstantial relative to the effect from an increase in usage [31]. Antimicrobial stewardship interventions have been shown to effectively reduce AMR, particularly when coupled with infection prevention and control measures [32].

This study has additional limitations that should be acknowledged. First, IRR estimates of the immediate level decreases observed for subgroups of specific pathogens immediately after the beginning of the intervention were too uncertain (too wide 95%CIs) due to the relatively small sample sizes in the subgroups. Second, the surveillance systems on which the study was based did not routinely record results of the molecular typing of isolated strains; thereby, the source of infection could not be traced further. Finally, our local setting is typical of tertiary hospital care provision in Greece, but there may be substantial variations within the country in clinical practice patterns, patient characteristics, microbial ecology and AMR, as well as varying levels of IPC implementation across hospitals in different regions. Therefore, the observed beneficial impact from enhanced infection prevention and behavioral precautions adopted during the COVID-19 period in this study may not be fully generalizable to other institutions and/or clinical settings.

In conclusion, this study demonstrates a collateral benefit of implementing SARS-CoV-2 preventive measures in two Greek hospitals, emphasizing that remaining focused on, building capacity in and enhancing routine IPC measures is a highly effective way to combat AMR and reduce, rapidly and substantially, the occurrence of difficult-to-treat HAIs from antibiotic-resistant ESKAPEE pathogens.

## 4. Materials and Methods

### 4.1. Study Design

This was a quasi-experimental study with an ITS design [18,19,20,21] to examine changes in levels and trends of the incidence of antibiotic-resistant ESKAPEE-associated HAIs before and after the natural experiment of applying enhanced prevention precautions during the COVID-19 period. To this end, clinical and microbiological data for all inpatients with confirmed HAIs caused by antibiotic-resistant ESKAPEE pathogens were examined and compared in two tertiary-care hospitals between March 2019 and February 2020 (pre-COVID-19, pre-intervention period) and between March 2020 and February 2021 (COVID-19 period, during the intervention). 

### 4.2. Setting

The two study centers were a 750-bed university-affiliated hospital (Hospital A) and a 440-bed general hospital (Hospital B), located in the island of Crete in Greece. Over the 24-month study period, the two hospitals cared for 110,032 and 69,597 inpatients (excluding day cases) for 353,792 and 187,453 patient-days, respectively. Both hospitals offer specialized services for surgical and medical patients, including critical care units. Hospital A offers additional and highly specialized services, including cardiothoracic surgery, surgical oncology, rheumatology and a pediatric ICU. Both hospitals receive referrals from other secondary and primary care centers on the island of Crete and nearby islands. Dedicated IPC teams, comprising an infectious disease physician, a clinical microbiologist and specialized nurses, oversee infection surveillance, prevention and control activities in these hospitals.

### 4.3. Eligible Patients and Sample Size

The study cohort included all patients with HAI caused by either VRE, MRSA, CR *K. pneumoniae*, CR *A. baumannii*, CR *P. aeruginosa*, CR *Enterobacter* spp. or CR *Escherichia coli*. These patients were prospectively identified by the active daily detection of nosocomial infections as part of established surveillance systems in the study hospitals. HAIs were defined according to the case definitions of the European Centre for Disease Prevention and Control [33]. 

The number of infections that occurred over the 24-month study period determined the size of the study, and no a priori statistical calculation of sample size was performed. Nevertheless, the study complied with the recommended minimum number of observations for ITS designs of 12 time points before and 12 time points after the intervention [20].

### 4.4. Data Collection

The following data were recorded for each patient: age, sex, ICD-10 codes for conditions documented on admission and during hospitalization, the date of first positive culture and microorganism(s) isolated, the department at the time of the first positive culture, results from antibiotic susceptibility tests, dates of admission and discharge and patient outcome (classified as discharged alive or inpatient death). To measure the severity of comorbid conditions, the Charlson comorbidity index (CCI) was calculated from ICD-10 codes using methods previously described [34].

### 4.5. Microbiology

Isolates were identified to the species level using standard biochemical methods and the Vitek 2 automated system (BIOMERIEUX). The latter was also used for susceptibility testing in Hospital A, whereas the Microscan WalkAway plus System (Beckman Coulter) was used in Hospital B. Resistance to carbapenems (meropenem or imipenem) was defined based on the breakpoints of the Clinical and Laboratory Standards Institute, through the detection of carbapenemase production using boronic acid and ethylene diamine-tetra-acetic acid combined with a carbapenem in disk test format or by detecting genes encoding the production of carbapenemases based on the modified Hodge test. E tests were used for the susceptibility testing of enterococci to vancomycin (breakpoint 4 mg/l). *S. aureus* isolates were phenotypically classified as MRSA based on the automated system (Vitek or Microscan) or using the cefoxitin disk diffusion test.

### 4.6. Interventions

Before the COVID-19 period, both hospitals implemented IPC programs incorporating the routine training of personnel in the ICUs, annual audits of hand hygiene practices [27] and, according to risk assessment, audits of contact and isolation precautions, as well as surveys of the cleaning and disinfection of surfaces and furnishings in the rooms of patients infected or colonized by antibiotic-resistant EKAPEE pathogens. Both hospitals implemented active daily surveillance of HAIs for tracer resistance phenotypes across all wards and departments and active colonization screening on ICU admission of high-risk patients. Patient isolation or cohorting were applied when possible to prevent the cross-transmission of multidrug-resistant pathogens. In March 2020, whilst anticipating admissions of COVID-19 patients, the IPC teams in both hospitals organized massive training activities on preventive measures (transmission-based precautions, hand hygiene and donning and doffing techniques for appropriate PPE usage) for all hospital staff, with intense training activities for healthcare workers who were most likely to manage COVID-19 cases. In addition, Hospital A’s IPC team was reinforced with two additional specialized nurses. During the COVID-19 period, both hospitals enforced the use of PPE, together with a stronger emphasis on hand hygiene. All operators wore surgical masks and/or FFP-2 filtering masks according to risk assessment, and the frequency and duration of the cleaning of patient rooms and surfaces were intensified. The number of family members and visitors was limited to the minimum possible, whereas access to ICUs and COVID-19 wards was restricted to healthcare professionals only. Moreover, non-essential elective surgical operations were reduced to compensate for the burden of COVID-19 admissions. Emergency department turnover was also reduced substantially during the lockdown periods (from 22 March to 4 May and from 7 November to 14 December 2020) when all non-essential movement was restricted throughout the country. Furthermore, annual leave for all medical and nursing staff was revoked to ensure an adequate workforce was on duty during the COVID-19 period.

Of note, Hospital A successfully implemented a carbapenem-focused antimicrobial stewardship program between January 2020 and December 2020, which led to improved prescribing and the reduced overall consumption of carbapenems in that hospital [30]. The policy for antibiotic use remained unchanged in Hospital B during the entire study. The full list of the interventions carried out in each hospital is included in Supplementary Table S1.

### 4.7. Outcomes

The primary study outcome was the rate of HAIs from antibiotic-resistant ESKAPEE pathogens. 

### 4.8. Statistical Methods

Overall changes in the epidemiology of infections were examined by comparing the clinical characteristics and outcomes of affected patients between the pre-COVID-19 (pre-intervention) period and the COVID-19 (intervention) period. Between-group differences were tested using the unpaired t-test for continuous data and Pearson’s chi-squared test for categorical data. Pooled infection incidence density rates (expressed per 1000 patient-days) were calculated over the two periods and were compared by means of incidence rate ratios (IRRs) with 95% confidence intervals (CIs) estimated with Poisson regression.

To investigate temporal changes in the incidence of antibiotic-resistant ESKAPEE HAIs (primary outcome) after the intervention was introduced accounting for underlying trends in the pre-intervention period, a segmented Poisson regression model for ITS analysis was applied on aggregated monthly incidence data [18,19,20,21]. This statistical approach allows the estimation of IRRs with 95%CIs to indicate level changes immediately after the beginning of the intervention and trend (slope) changes during the intervention period [18,19,20]. In the segmented regression model used for this study, the series of monthly counts of infections formed the dependent variable. Independent variables were the time elapsed since the start of the study, the intervention period indicator (post- vs. pre-March 2020) and the time after the intervention. The monthly series of patient-days (log transformed) was used as an offset variable to convert the outcome into an incidence rate that adjusts for potential variation in the size of the hospital population and LOS over time. A pair of sine–cosine Fourier functions of time was included to remove seasonal variation. The model coefficients were estimated using the maximum likelihood method. Residual autocorrelation was ruled out by examining correlograms and the Durbin–Watson test. 

Subgroup analyses were performed using the site of infection, pathogen species, source hospital and clinical service (ICU vs. ordinary wards). None of the study variables had missing data. Statistical significance was indicated at the conventional *p* < 0.05 threshold. All analyses were performed using Stata version 18 (Stata Corp., College Station, TX, USA).

### 4.9. Ethics and Reporting

The study was approved by the Institutional Ethics Review Boards of the participating hospitals (approvals 6/12-01-2023 and 130/22-02-2023) and the 7th Regional Health Authority of Greece (approval no. 22858). The study is reported in accordance with the Transparent Reporting of Evaluations with Nonrandomized Designs (TREND) guidelines [35].

## Figures and Tables

**Table 1:** Demographics, clinical characteristics and outcomes of patients with documented healthcare-associated infection(s) caused by multidrug-resistant ESKAPEE pathogens.

Variable	Pre-COVID-19	COVID-19 Period			
	All Patients (*n* = 235) ^a^	All Patients (*n* = 204) ^a^	*p* ^b^	Non-COVID-19 Patients (*n* = 184) ^a^	*p* ^b^
Hospital			0.15		0.13
A	173 (74%)	162 (79%)		147 (80%)	
B	62 (26%)	42 (21%)		37 (20%)	
Age (years)	60.0 ± 22.1	66.0 ± 18.2	0.002	65.2 ± 18.7	0.010
Male sex	159 (68%)	133 (65%)	0.59	120 (65%)	0.60
Severe COVID-19 patients	0 (0%)	20 (10%)	<0.001	0 (0%)	
Reason for hospital admission			0.49		0.96
Circulatory system disease	41 (17%)	37 (18%)		37 (20%)	
Injury, poisoning, external cause	34 (14%)	27 (13%)		27 (15%)	
Respiratory system disease	36 (15%)	25 (12%)		25 (14%)	
Neoplasm	31 (13%)	24 (12%)		24 (13%)	
Symptom, sign, abnormal finding	31 (13%)	20 (10%)		19 (10%)	
Digestive system disease	16 (7%)	14 (7%)		14 (8%)	
Other disease or condition	46 (20%)	57 (28%)		38 (21%)	
Charlson comorbidity index			0.39		0.19
0	160 (68%)	134 (66%)		115 (62%)	
1	35 (15%)	40 (20%)		40 (22%)	
2+	40 (17%)	30 (15%)		29 (16%)	
Department at time of index infection			0.82		0.24
Intensive care unit	105 (45%)	84 (41%)		65 (35%)	
Medical ward	81 (34%)	71 (35%)		70 (38%)	
Surgical ward	44 (19%)	45 (22%)		45 (24%)	
Pediatric or obstetrics ward	5 (2%)	4 (2%)		4 (2%)	
Pre-index infection LOS (days)	18.2 ± 16.8	22.0 ± 22.7	0.043	21.8 ± 23.4	0.068
Infection status			0.82		0.97
Single infection	183 (78%)	157 (77%)		143 (78%)	
Multiple infections	52 (22%)	47 (23%)		41 (22%)	
Polymicrobial infection	33 (14%)	16 (8%)	0.040	15 (8%)	0.060
14-day outcome ^c^			0.14		0.27
Discharged alive	47 (20%)	34 (17%)		34 (18%)	
Remain hospitalized	148 (63%)	120 (59%)		107 (58%)	
Died in hospital	40 (17%)	50 (25%)		43 (23%)	
In-hospital mortality			0.21		0.68
Discharged alive	135 (57%)	105 (51%)		102 (55%)	
Died in hospital	100 (43%)	99 (49%)		82 (45%)	
Overall LOS (days)	51.4 ± 47.8	45.8 ± 38.0	0.18	45.7 ± 39.3	0.19

LOS, length of hospital stay. ^a^ Data are presented as mean ± SD for continuous variables, and n (%) of patients for categorical variables. ^b^ *p* values refer to the comparison of the two patient groups in the pre-COVID-19 and COVID-19 periods. ^c^ Within 14 days from the onset of the first infection for patients with multiple consecutive infections.

**Table 2:** Types and microbiology of healthcare-associated infection episodes (*n* = 586) caused by multidrug-resistant ESKAPEE pathogens.

Subgroup	Pre-COVID-19	COVID-19 Period			
	All Infection Episodes (*n* = 311) ^a,b^	All Infection Episodes (*n* = 275) ^a,b^	*p* ^c^	Infection Episodes in Non-COVID-19 Patients (*n* = 249) ^a,b^	*p* ^c^
Infection site					
Bloodstream infection	131 (42%)	128 (47%)	0.28	116 (47%)	0.29
Intubation-associated pneumonia	26 (8%)	31 (11%)	0.24	22 (9%)	0.84
Hospital-acquired pneumonia	20 (6%)	40 (15%)	0.001	37 (15%)	0.001
Lower respiratory tract infection	65 (21%)	55 (20%)	0.79	50 (20%)	0.81
Surgical site infection	32 (10%)	32 (12%)	0.60	32 (13%)	0.34
Urinary tract infection	25 (8%)	15 (5%)	0.22	15 (6%)	0.36
Skin and soft-tissue infection	8 (3%)	2 (1%)	0.085	2 (1%)	0.12
Other type of infection	12 (4%)	3 (1%)	0.034	3 (1%)	0.053
Polymicrobial infections	35 (11%)	18 (7%)	0.047	17 (7%)	0.073
Pathogen					
VRE	27 (9%)	30 (11%)	0.36	30 (12%)	0.19
MRSA	25 (8%)	33 (12%)	0.11	30 (12%)	0.11
CR *Klebsiella pneumoniae*	39 (13%)	21 (8%)	0.051	19 (8%)	0.058
CR *Acinetobacter baumannii*	183 (59%)	166 (60%)	0.71	147 (59%)	0.96
CR *Pseudomonas aeruginosa*	69 (22%)	38 (14%)	0.009	35 (14%)	0.014
CR *Enterobacter* spp.	1 (0%)	7 (3%)	0.021	7 (3%)	0.014
CR *Escherichia coli*	3 (1%)	0 (0%)	0.10	0 (0%)	0.12

VRE, vancomycin-resistant *Enterococcus faecium* or *Enterococcus faecalis*; MRSA, methicillin-resistant *Staphylococcus aureus*; CR, carbapenem-resistant. ^a^ Data are presented as mean ± SD for continuous variables, and n (%) for categorical variables. ^b^ Sums of reported percentages for implicated infections sites and infecting organisms exceed 100% due to some patients having multiple infections at different sites and polymicrobial infections. ^c^ *p* values refer to the comparison of the two groups in the pre-COVID-19 and COVID-19 periods.

## Data Availability

Anonymized and aggregated time series data are available on reasonable request from the corresponding author.

## Supplementary Materials

The following supporting information can be downloaded at: https://www.mdpi.com/article/10.3390/antibiotics12071088/s1, Table S1: Interventions for infection prevention and control that were implemented during the 12 months prior to and the first 12 months during the COVID-19 pandemic at the two study hospitals. Table S2: Clinical characteristics and outcomes of patients with documented healthcare-associated infections caused by multidrug-resistant ESKAPEE pathogens. Table S3: Stratified analysis by source hospital of clinical characteristics and outcomes in patients with documented healthcare-associated infection caused by multidrug-resistant ESKAPEE pathogens. Table S4: Stratified analysis by source hospital of the sites and microbiology of healthcare-associated infection episodes caused by multidrug-resistant ESKAPEE pathogens. Table S5: Comparison of pooled (averaged) incidence rates of healthcare-associated infections from multidrug-resistant ESKAPE pathogens (per 1000 patient-days) over the 12 months before and the 12 months during the COVID-19 pandemic. Table S6: Estimated level changes in the incidence of antibiotic-resistant ESKAPEE healthcare-associated infections (per 1000 patient-days) occurring immediately after introducing enhanced infection prevention and control measures. Figure S1: Monthly rates of healthcare-associated infections from multidrug-resistant ESKAPEE pathogens, before and during the intervention, presented separately for intensive care units and ordinary wards.
