# Supplementary material for: Impact of SARS-CoV-2 Preventive Measures against Healthcare-Associated Infections from Antibiotic-Resistant ESKAPEE Pathogens: A Two-Center, Natural Quasi-Experimental Study in Greece
Source: Antibiotics (Basel). 2023 Jun 22;12(7):1088. doi: 10.3390/antibiotics12071088 (PMC10376605; doi:10.3390/antibiotics12071088)
Supplement: Supplementary file 1 [file antibiotics-12-01088-s001.zip › antibiotics-2447911-supplementary.pdf]

SUPPLEMENTARY MATERIALS

Impact of SARS-CoV-2 Preventive Measures against Healthcare-Associated Infections from Antibiotic-Resistant ESKAPEE Pathogens: A Two-Center, Natural Quasi-Experimental Study in Greece

Table S1. Interventions for infection prevention and control that were implemented during the 12 months prior to and the first 12 months during the COVID-19 pandemic at the two study hospitals

Brief description of intervention component or activity	Hospital A (750 beds)	Hospital B (440 beds)
Pre-COVID-19 period (March 2019 to February 2020), standard measures:		
Dedicated team overseeing all IPC activities (no. of specialized nurses)	✓ (1)	✓ (1)
Routine IPC training of hospital staff in ICUs	✓	✓
Annual audits of hand hygiene compliance in wards and ICUs	✓	✓
Audits of contact and isolation precautions according to risk-assessment	✓	✓
Active daily surveillance of MDR HAIs	✓	✓
Active colonization screening of high-risk patients on ICU admission	✓	✓
Patient isolation or cohorting to prevent cross-transmission of MDR pathogens	✓	✓
Surface and furnishing disinfection in rooms of patients with MDR EKAPEE isolates	✓	✓
COVID-19 period (March 2020 to February 2021), additional or enhanced measures:		
Additional specialized nurses joining the IPC team (number added)	✓ (+2)	✘
IPC training expanded for all hospital staff and intensified in high-risk settings	✓	✓
Enforced PPE and hand hygiene protocols	✓	✓
Increased frequency and duration of cleaning of patient rooms and surfaces	✓	✓
All operators required to wear surgical masks and/or FFP-2 filtering masks	✓	✓
Reduced number and delaying of non-essential elective surgeries	✓	✓
Reduced emergency department visits (due to lockdowns)	✓	✓
Strict hospital visitation restrictions, limited access to ICU	✓	✓
Annual leave for all medical and nursing staff revoked	✓	✓
New antimicrobial stewardship intervention	✓ *	✘

IPC, infection prevention and control; ICU, intensive care unit; MDR, multidrug resistant; PPE, personal protective equipment.

***** A carbapenem-focused antimicrobial stewardship program was implemented in Hospital A between January 2020 and December 2020.

Table S2. Clinical characteristics and outcomes of patients with documented healthcare-associated infections caused by antibiotic resistant ESKAPEE pathogens

**Variable**	**Total (*n* = 439)** [a]	**Hospital A (*n* = 335)** [a]	**Hospital B (*n* = 104)** [a]	***p*** [b]
Age (years)	62.8±20.6	62.5±21.3	63.6±18.4	0.64
Male sex	292 (67%)	217 (65%)	75 (72%)	0.17
Severe COVID-19 patient	20 (5%)	15 (4%)	5 (5%)	0.89
Reason for hospital admission (ICD-10 code)				0.043
Circulatory system disease (I00–I99)	78 (18%)	62 (19%)	16 (15%)	
Injury, poisoning, external cause (S00–T98)	61 (14%)	47 (14%)	14 (13%)	
Respiratory system disease (J00–J99)	61 (14%)	45 (13%)	16 (15%)	
Neoplasm (C00–D48)	55 (13%)	46 (14%)	9 (9%)	
Symptom, sign, abnormal finding (R00–R99)	51 (12%)	31 (9%)	20 (19%)	
Digestive system disease (K00–K93)	30 (7%)	28 (8%)	2 (2%)	
COVID-19 (U07)	18 (4%)	14 (4%)	4 (4%)	
Other disease or condition	85 (19%)	62 (19%)	23 (22%)	
Charlson comorbidity index				0.90
0	294 (67%)	225 (67%)	69 (66%)	
1	75 (17%)	58 (17%)	17 (16%)	
2+	70 (16%)	52 (16%)	18 (17%)	
Department at time of index infection				0.11
Intensive care unit	189 (43%)	147 (44%)	42 (40%)	
Medical ward	152 (35%)	108 (32%)	44 (42%)	
Surgical ward	89 (20%)	71 (21%)	18 (17%)	
Paediatric or obstetrics ward	9 (2%)	9 (3%)	0 (0%)	
Pre-index infection LOS (days)	20.0±19.9	20.6±21.1	17.8±15.0	0.21
Infection status				<0.001
Single infection	340 (77%)	242 (72%)	98 (94%)	
Multiple infections	99 (23%)	93 (28%)	6 (6%)	
Polymicrobial infection	49 (11%)	38 (11%)	11 (11%)	0.83
14-day outcome [c]				0.83
Discharged alive	81 (18%)	60 (18%)	21 (20%)	
Remain hospitalized	268 (61%)	207 (62%)	61 (59%)	
Died in hospital	90 (21%)	68 (20%)	22 (21%)	
In-hospital mortality				0.19
Discharged alive	240 (55%)	189 (56%)	51 (49%)	
Died in hospital	199 (45%)	146 (44%)	53 (51%)	
Overall LOS (days)	48.8±43.6	50.3±45.0	44.1±38.6	0.21

LOS, length of stay.
[a] Data are presented as mean ± SD for continuous variables, and n (%) of patients for categorical variables.
[b] *p* values refer to the comparison of the two patient groups in the pre-COVID-19 and Covid-19 periods
[c] Within 14 days from the onset of the first infection for patients with multiple consecutive infections.

Table S3. Stratified analysis by source hospital of clinical characteristics and outcomes in patients with documented healthcare-associated infection caused by multidrug-resistant ESKAPEE pathogens

	Hospital A			Hospital B		
**Variable**	**Pre-COVID-19 (*n* = 173)** [a]	**COVID-19 (*n* = 162)** [a]	***p*** [b]	**Pre-COVID-19 (*n* = 62)** [a]	**COVID-19 (*n* = 42)** [a]	***p*** [b]
Age (years)	59.6±23.0	65.7±18.8	0.009	61.0±19.7	67.5±15.6	0.076
Male sex	112 (65%)	105 (65%)	0.99	47 (76%)	28 (67%)	0.31
Severe COVID-19 patients	0 (0%)	15 (9%)	<0.001	0 (0%)	5 (12%)	0.005
Reason for hospital admission (ICD-10 code)			0.28			0.98
Circulatory system disease (I00–I99)	32 (18%)	30 (19%)		9 (15%)	7 (17%)	
Injury, poisoning, external cause (S00–T98)	24 (14%)	23 (14%)		10 (16%)	4 (10%)	
Respiratory system disease (J00–J99)	27 (16%)	18 (11%)		9 (15%)	7 (17%)	
Neoplasm (C00–D48)	26 (15%)	20 (12%)		5 (8%)	4 (10%)	
Symptom, sign, abnormal finding (R00–R99)	19 (11%)	12 (7%)		12 (19%)	8 (19%)	
Digestive system disease (K00–K93)	15 (9%)	13 (8%)		1 (2%)	1 (2%)	
Other disease or condition	30 (17%)	46 (28%)		16 (26%)	11 (26%)	
Charlson comorbidity index			0.40			0.72
0	117 (68%)	108 (67%)		43 (69%)	26 (62%)	
1	26 (15%)	32 (20%)		9 (15%)	8 (19%)	
2+	30 (17%)	22 (14%)		10 (16%)	8 (19%)	
Department at time of index infection			0.78			0.88
Intensive care unit	79 (46%)	68 (42%)		26 (42%)	16 (38%)	
Medical ward	56 (32%)	52 (32%)		25 (40%)	19 (45%)	
Surgical ward	33 (19%)	38 (23%)		11 (18%)	7 (17%)	
Pediatric or obstetrics ward	5 (3%)	4 (2%)		0 (0%)	0 (0%)	
Pre-index infection LOS (days)	18.4±17.7	23.0±24.1	0.049	17.5±14.2	18.3±16.2	0.78
Infection status			0.80			0.22
Single infection	126 (73%)	116 (72%)		57 (92%)	41 (98%)	
Multiple infections	47 (27%)	46 (28%)		5 (8%)	1 (2%)	
Polymicrobial infection	24 (14%)	14 (9%)	0.13	9 (15%)	2 (5%)	0.11
14-day outcome [c]			0.44			0.13
Discharged alive	34 (20%)	26 (16%)		13 (21%)	8 (19%)	
Remain hospitalized	108 (62%)	99 (61%)		40 (65%)	21 (50%)	
Died in hospital	31 (18%)	37 (23%)		9 (15%)	13 (31%)	
In-hospital mortality			0.33			0.30
Discharged alive	102 (59%)	87 (54%)		33 (53%)	18 (43%)	
Died in hospital	71 (41%)	75 (46%)		29 (47%)	24 (57%)	
Overall LOS (days)	51.9±49.0	48.5±40.4	0.49	50.1±44.9	35.3±24.7	0.055

LOS, length of stay.
[a] Data are presented as mean ± SD for continuous variables, and n (%) of patients for categorical variables.
[b] *p* values refer to the comparison of the two patient groups in the pre-COVID-19 and Covid-19 periods in each hospital.
[c] Within 14 days from the onset of the first infection for patients with multiple consecutive infections.

Table S4. Stratified analysis by source hospital of the sites and microbiology of healthcare-associated infection episodes caused by multidrug-resistant ESKAPEE pathogens

	Hospital A			Hospital B		
**Subgroup**	**Pre-COVID-19 (*n* = 242)** [a,b]	**COVID-19 (*n* = 232)** [a,b]	***p*** [c]	**Pre-COVID-19 (*n* = 69)** [a,b]	**COVID-19 (*n* = 43)** [a,b]	***p*** [c]
Infection site						
Bloodstream infection	106 (44%)	100 (43%)	0.88	25 (36%)	28 (65%)	0.003
Intubation-associated pneumonia	21 (9%)	28 (12%)	0.23	5 (7%)	3 (7%)	0.96
Hospital-acquired pneumonia	12 (5%)	37 (16%)	<0.001	8 (12%)	3 (7%)	0.42
Lower respiratory tract infection	38 (16%)	46 (20%)	0.24	27 (39%)	9 (21%)	0.045
Surgical site infection	28 (12%)	31 (13%)	0.55	4 (6%)	1 (2%)	0.39
Urinary tract infection	18 (7%)	14 (6%)	0.54	7 (10%)	1 (2%)	0.12
Skin and soft-tissue infection	8 (3%)	1 (0%)	0.022	0 (0%)	1 (2%)	0.20
Other type of infection	11 (5%)	3 (1%)	0.037	1 (1%)	0 (0%)	0.43
Polymicrobial infection	26 (11%)	16 (7%)	0.14	9 (13%)	2 (5%)	0.15
Pathogen						
VRE	20 (8%)	24 (10%)	0.44	7 (10%)	6 (14%)	0.54
MRSA	19 (8%)	28 (12%)	0.12	6 (9%)	5 (12%)	0.61
CR *Klebsiella pneumoniae*	35 (14%)	20 (9%)	0.047	4 (6%)	1 (2%)	0.39
CR *Acinetobacter baumannii*	132 (55%)	144 (62%)	0.097	51 (74%)	22 (51%)	0.014
CR *Pseudomonas aeruginosa*	59 (24%)	27 (12%)	<0.001	10 (14%)	11 (26%)	0.14
CR *Enterobacter* spp.	1 (0%)	7 (3%)	0.028	0 (0%)	0 (0%)	-
CR *Escherichia coli*	3 (1%)	0 (0%)	0.089	0 (0%)	0 (0%)	-

VRE, vancomycin-resistant *Enterococcus faecium* or *Enterococcus faecalis*; MRSA, methicillin-resistant *Staphylococcus aureus*; CR, carbapenem-resistant.

[a] Data are presented as mean ± SD for continuous variables, and n (%) for categorical variables.

[b] Sums of reported percentages for implicated infections sites and infecting organisms exceed 100% due to some patients having multiple infections at different sites and polymicrobial infections.

[c] *p* values refer to the comparison of the two groups in the pre-COVID-19 and Covid-19 periods in each hospital.

Table S5. Comparison of pooled (averaged) incidence rates of healthcare-associated infections from multidrug resistant ESKAPE pathogens (per 1000 patient-days) over the 12 months before and the 12 months during the COVID-19 pandemic

**Group**	**Hospital A**		**Hospital B**		**Total**	
	**IRR (95% CI)**	***p***	**IRR (95% CI)**	***p***	**IRR (95% CI)**	***p***
Infected patients	1.08 (0.86–1.34)	0.49	0.85 (0.56–1.27)	0.40	1.03 (0.85–1.24)	0.78
Infections	1.10 (0.92–1.33)	0.28	0.78 (0.52–1.15)	0.20	1.05 (0.89–1.23)	0.58
By site:						
Bloodstream infections	1.09 (0.82–1.44)	0.56	1.40 (0.79–2.50)	0.23	1.16 (0.90–1.49)	0.24
Intubation-associated pneumonias	1.53 (0.84–2.84)	0.14	0.75 (0.12–3.85)	0.72	1.41 (0.81–2.47)	0.20
Hospital-acquired pneumonias	3.55 (1.81–7.48)	< 0.001	0.47 (0.08–1.95)	0.27	2.37 (1.35–4.27)	0.001
Lower respiratory tract infections	1.39 (0.89–2.20)	0.13	0.42 (0.17–0.91)	0.018	1.00 (0.69–1.46)	0.99
Surgical site infections	1.27 (0.74–2.20)	0.35	0.31 (0.01–3.15)	0.32	1.18 (0.70–2.00)	0.50
Urinary tract infections	0.90 (0.41–1.91)	0.76	0.18 (0.00–1.39)	0.076	0.71 (0.35–1.40)	0.30
Skin and soft-tissue infections	0.14 (0.00–1.07)	0.035	ne	0.45	0.30 (0.03–1.48)	0.11
Other type of infections	0.31 (0.06–1.19)	0.063	0.00 (0.00–48.66)	0.56	0.30 (0.05–1.10)	0.046
By pathogen:						
VRE	1.38 (0.73–2.64)	0.29	1.07 (0.30–3.72)	0.90	1.31 (0.76–2.30)	0.30
MRSA	1.70 (0.91–3.21)	0.075	1.04 (0.25–4.09)	0.94	1.56 (0.90–2.74)	0.093
CR *Klebsiella pneumoniae*	0.66 (0.36–1.17)	0.13	0.31 (0.01–3.15)	0.32	0.64 (0.36–1.11)	0.094
CR *Acinetobacter baumannii*	1.26 (0.98–1.60)	0.059	0.54 (0.31–0.90)	0.013	1.07 (0.86–1.33)	0.51
CR *Pseudomonas aeruginosa*	0.53 (0.32–0.84)	0.005	1.37 (0.53–3.60)	0.48	0.65 (0.43–0.98)	0.032

IRR, incidence rate ratio, CI, confidence interval; ne, non-estimable.

Table S6. Estimated level changes in the incidence of antibiotic resistant ESKAPEE healthcare-associated infections (per 1000 patient-days) occurring immediately after introducing enhanced infection prevention and control measures

Group	Hospital A		Hospital B		Total	
	**Level change IRR (95% CI)**	***p***	**Level change IRR (95% CI)**	***p***	**Level change IRR (95% CI)**	***p***
All Infections combined	0.55 (0.31–0.98)	0.042	0.49 (0.14–1.64)	0.24	0.55 (0.33–0.93)	0.027
By site:						
Bloodstream infections	0.67 (0.27–1.66)	0.39	0.40 (0.07–2.33)	0.31	0.61 (0.27–1.35)	0.22
Intubation-associated pneumonias	0.25 (0.05–1.19)	0.081	ne	-	0.52 (0.13–2.12)	0.36
Hospital-acquired pneumonias	0.11 (0.01–1.65)	0.11	0.52 (0.01–24.93)	0.74	0.17 (0.02–1.37)	0.096
Lower respiratory tract infections	0.61 (0.15–2.50)	0.50	0.12 (0.01–1.86)	0.13	0.46 (0.14–1.51)	0.20
Surgical site infections	0.72 (0.12–4.42)	0.72	0.01 (0.00–738.81)	0.42	0.67 (0.11–4.01)	0.67
Urinary tract infections	1.12 (0.11–11.13)	0.93	ne	-	1.60 (0.19–13.76)	0.67
Skin and soft-tissue infections	0.13 (0.00–21.54)	0.43	ne	-	0.18 (0.00–20.76)	0.47
Other type of infections	2.79 (0.06–122.79)	0.59	ne	-	1.72 (0.05–57.02)	0.76
By pathogen:						
VRE	3.16 (0.31–31.75)	0.33	0.59 (0.01–41.40)	0.81	2.47 (0.35–17.41)	0.36
MRSA	0.39 (0.06–2.75)	0.34	0.02 (0.00–3.94)	0.14	0.24 (0.04–1.47)	0.12
CR *Klebsiella pneumoniae*	0.11 (0.02–0.61)	0.012	0.21 (0.00–747.60)	0.71	0.14 (0.03–0.69)	0.016
CR *Acinetobacter baumannii*	0.72 (0.35–1.49)	0.38	0.36 (0.08–1.60)	0.18	0.65 (0.34–1.24)	0.19
CR *Pseudomonas aeruginosa*	0.37 (0.08–1.81)	0.22	0.16 (0.00–5.94)	0.32	0.33 (0.08–1.37)	0.13

IRR, incidence rate ratio, CI, confidence interval; ne, non-estimable

Figure S1. Monthly rates of healthcare-associated infections from multidrug-resistant ESKAPEE pathogens, before and during the intervention, presented separately for intensive care units and ordinary wards. Dots: observed rates. Solid line: predicted rates from Poisson regression model adjusted for seasonality. Dashed line: deseasonalized trend. Vertical long dashed line: time of the beginning of the intervention.

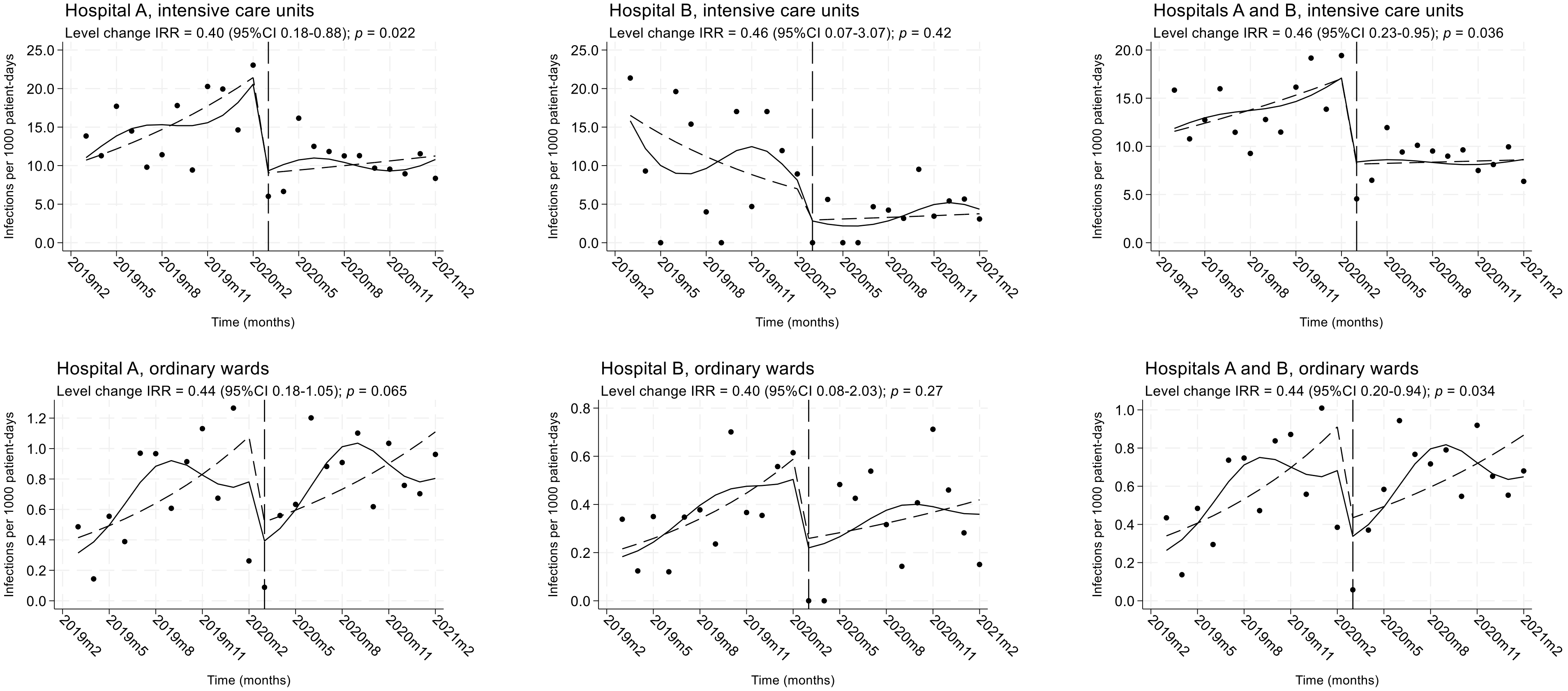

TREND Statement Checklist

Paper Section/Topic	Item No.	Descriptor	Reported? ✓	Reported? Section
**TITLE and ABSTRACT**				
Title and Abstract	1	• Information on how units were allocated to interventions	✓	Title and Abstract
		• Structured abstract recommended	✓	Abstract
		• Information on target population or study sample	✓	Abstract
**INTRODUCTION**				
Background	2	• Scientific background and explanation of rationale	✓	1. Introduction
		• Theories used in designing behavioral interventions	N/A	-
**METHODS**				
Participants	3	• Eligibility criteria for participants, including criteria at different levels in recruitment/sampling plan (e.g., cities, clinics, subjects)	✓	4.3 Eligible patients and sample size
		• Method of recruitment (e.g., referral, self-selection), including the sampling method if a systematic sampling plan was implemented	✓	4.1 Study design
		• Recruitment setting	✓	4.2 Setting
		• Settings and locations where the data were collected	✓	4.2 Setting
Interventions	4	• Details of the interventions intended for each study condition and how and when they were actually administered, specifically including:	✓	4.6 Interventions, Table S1
		○ Content: what was given?	✓	4.6 Interventions, Table S1
		○ Delivery method: how was the content given?	✓	4.6 Interventions
		○ Unit of delivery: how were subjects grouped during delivery?	✓	4.6 Interventions
		○ Deliverer: who delivered the intervention?	✓	4.6 Interventions
		○ Setting: where was the intervention delivered?	✓	4.6 Interventions, 4.2 Setting
		○ Exposure quantity and duration: how many sessions or episodes or events were intended to be delivered? How long were they intended to last?	N/A	-
		○ Time span: how long was it intended to take to deliver the intervention to each unit?	N/A	-
		○ Activities to increase compliance or adherence (e.g., incentives)	✓	4.6 Interventions
Objectives	5	• Specific objectives and hypotheses	✓	1. Introduction
Outcomes	6	• Clearly defined primary and secondary outcome measures	✓	4.7 Outcomes
		• Methods used to collect data and any methods used to enhance the quality of measurements	✓	4.4 Data collection
		• Information on validated instruments such as psychometric and biometric properties	N/A	-
Sample size	7	• How sample size was determined and, when applicable, explanation of any interim analyses and stopping rules	✓	4.3 Eligible patients and sample size
Assignment	8	• Unit of assignment (the unit being assigned to study condition, e.g., individual, group, community)	✓	4.6 Interventions

method		• Method used to assign units to study conditions, including details of any restriction (e.g., blocking, stratification, minimization)	✓	4.6 Interventions
		• Inclusion of aspects employed to help minimize potential bias induced due to non-randomization (e.g., matching)	N/A	-
Blinding (masking)	9	• Whether or not participants, those administering the interventions, and those assessing the outcomes were blinded to study condition assignment; if so, statement regarding how the blinding was accomplished and how it was assessed	N/A	-
Unit of Analysis	10	• Description of the smallest unit that is being analysed to assess intervention effects (e.g., individual, group, or community)	✓	4.6 Interventions, 4.8 Statistical methods
		• If the unit of analysis differs from the unit of assignment, the analytical method used to account for this (e.g., adjusting the standard error estimates by the design effect or using multilevel analysis)	N/A	-
Statistical methods	11	• Statistical methods used to compare study groups for primary methods outcome(s), including complex methods for correlated data	✓	4.8 Statistical methods
		• Statistical methods used for additional analyses, such as subgroup analyses and adjusted analysis	✓	4.8 Statistical methods
		• Methods for imputing missing data, if used	N/A	-
		• Statistical software or programs used	✓	4.8 Statistical methods
**RESULTS**				
Participant flow	12	• Flow of participants through each stage of the study: enrollment, assignment, allocation and intervention exposure, follow-up, analysis (a diagram is strongly recommended)	N/A	-
		○ Enrollment: the numbers of participants screened for eligibility, found to be eligible or not eligible, declined to be enrolled, and enrolled in the study	N/A	-
		○ Assignment: the numbers of participants assigned to a study condition	✓	Table 1
		○ Allocation and intervention exposure: the number of participants assigned to each study condition and the number of participants who received each intervention	✓	Table 1
		○ Follow-up: the number of participants who completed the follow-up or did not complete the follow-up (i.e., lost to follow-up), by study condition	N/A	-
		○ Analysis: the number of participants included in or excluded from the main analysis, by study condition	✓	Table 1
		• Description of protocol deviations from study as planned, along with reasons	N/A	-
Recruitment	13	• Dates defining the periods of recruitment and follow-up	✓	4.6 Interventions, Table S1
Baseline data	14	• Baseline demographic and clinical characteristics of participants in each study condition	✓	2.1 Affected patients, Table 1
		• Baseline characteristics for each study condition relevant to specific disease prevention research	N/A	-
		• Baseline comparisons of those lost to follow-up and those retained, overall and by study condition	N/A	-
		• Comparison between study population at baseline and target population of interest	✓	Tables & Figures
Baseline equivalence	15	• Data on study group equivalence at baseline and statistical methods used to control for baseline differences	✓	Tables & Figures
Numbers analyzed	16	• Number of participants (denominator) included in each analysis for each study condition, particularly when the denominators change for different outcomes; statement of the results in absolute numbers when feasible	✓	Tables & Figures
		• Indication of whether the analysis strategy was “intention to treat”	N/A	-

		or, if not, description of how non-compliers were treated in the analyses		
Outcomes and estimation	17	• For each primary and secondary outcome, a summary of results for each estimation study condition, and the estimated effect size and a confidence interval to indicate the precision	✓	2.2 Before-after pooled infection rates, 2.3 Temporal trends and level changes in infection rates, Tables & Figures
		• Inclusion of null and negative findings	N/A	-
		• Inclusion of results from testing pre-specified causal pathways through which the intervention was intended to operate, if any	N/A	-
Ancillary analyses	18	• Summary of other analyses performed, including subgroup or restricted analyses, indicating which are pre-specified or exploratory	✓	Results, 2.4 ICUs vs. wards, Supplement
Adverse events	19	• Summary of all important adverse events or unintended effects in each study condition (including summary measures, effect size estimates, and confidence intervals)	✓	Tables & Figures
**DISCUSSION**				
Interpretation	20	• Interpretation of the results, taking into account study hypotheses, sources of potential bias, imprecision of measures, multiplicative analyses, and other limitations or weaknesses of the study	✓	Discussion
		• Discussion of results taking into account the mechanism by which the intervention was intended to work (causal pathways) or alternative mechanisms or explanations	✓	Discussion
		• Discussion of the success of and barriers to implementing the intervention, fidelity of implementation	✓	Discussion
		• Discussion of research, programmatic, or policy implications	✓	Discussion
Generalizability	21	• Generalizability (external validity) of the trial findings, taking into account the study population, the characteristics of the intervention, length of follow-up, incentives, compliance rates, specific sites/settings involved in the study, and other contextual issues	✓	Discussion
Overall evidence	22	• General interpretation of the results in the context of current evidence and current theory	✓	Discussion
